# VEGF Over-Expression by Engineered BMSC Accelerates Functional Perfusion, Improving Tissue Density and In-Growth in Clinical-Size Osteogenic Grafts

**DOI:** 10.3389/fbioe.2020.00755

**Published:** 2020-07-03

**Authors:** Rene’ D. Largo, Maximilian G. Burger, Oliver Harschnitz, Conny F. Waschkies, Andrea Grosso, Celeste Scotti, Alexandre Kaempfen, Sinan Gueven, Gernot Jundt, Arnaud Scherberich, Dirk J. Schaefer, Andrea Banfi, Nunzia Di Maggio

**Affiliations:** ^1^Cell and Gene Therapy, Department of Biomedicine, >Basel University Hospital and University of Basel, Basel, Switzerland; ^2^Plastic and Reconstructive Surgery, Department of Surgery, Basel University Hospital and University of Basel, Basel, Switzerland; ^3^Institute for Biomedical Engineering, ETH and University of Zurich, Zurich, Switzerland; ^4^Department of Surgical Research, University Hospital Zurich, Zurich, Switzerland; ^5^Tissue Engineering, Department of Biomedicine, University Hospital of Basel, University of Basel, Basel, Switzerland; ^6^Institute of Pathology, University Hospital of Basel, Basel, Switzerland

**Keywords:** VEGF, bone marrow stromal cells, osteogenic grafts, vascularization, gene therapy

## Abstract

The first choice for reconstruction of clinical-size bone defects consists of autologous bone flaps, which often lack the required mechanical strength and cause significant donor-site morbidity. We have previously developed biological substitutes in a rabbit model by combining bone tissue engineering and flap pre-fabrication. However, spontaneous vascularization was insufficient to ensure progenitor survival in the core of the constructs. Here, we hypothesized that increased angiogenic stimulation within constructs by exogenous VEGF can significantly accelerate early vascularization and tissue in-growth. Bone marrow stromal cells from NZW rabbits (rBMSC) were transduced with a retroviral vector to express rabbit VEGF linked to a truncated version of rabbit CD4 as a cell-surface marker. Autologous cells were seeded in clinical-size 5.5 cm^3^ HA scaffolds wrapped in a panniculus carnosus flap to provide an ample vascular supply, and implanted ectopically. Constructs seeded with VEGF-expressing rBMSC showed significantly increased progenitor survivival, depth of tissue ingrowth and amount of mineralized tissue. Contrast-enhanced MRI after 1 week *in vivo* showed significantly improved tissue perfusion in the inner layer of the grafts compared to controls. Interestingly, grafts containing VEGF-expressing rBMSC displayed a hierarchically organized functional vascular tree, composed of dense capillary networks in the inner layers connected to large-caliber feeding vessels entering the constructs at the periphery. These data constitute proof of principle that providing sustained VEGF signaling, independently of cells experiencing hypoxia, is effective to drive rapid vascularization and increase early perfusion in clinical-size osteogenic grafts, leading to improved tissue formation deeper in the constructs.

## Introduction

Bone grafts are crucial to treat bone tissue loss due to trauma, surgery or other clinical conditions where physiological bone repair is insufficient. Autografts are currently the gold standard for bone repair, but this approach is often associated with several limitations, including chronic pain at the harvest site, neurovascular injury, structural weakness and the limited amount of autologous bone available (Younger and Chapman, 1989; Giladi et al., 2018). Moreover, upon implantation *in vivo*, a major hurdle, especially for large defects, is osteonecrosis due to lack of an endogenous vascular network which can improve host integration (Rouwkema et al., 2008). Grafts consisting of bone tissue and an internal vascular network supplied by large caliber vessels, called bone flaps, have been applied for surgical reconstruction of large segmental defects (Sparks et al., 2017). However, bone flaps also suffer from complications, including insufficient mechanical strength, significant donor site morbidity, as well as complicated surgical procedures to generate grafts of a predefined shape (Bodde et al., 2003). Therefore, available surgical strategies are currently insufficient and there is an unmet clinical need to promote vascularized bone regeneration. Bone tissue-engineering holds promise for the generation of osteogenic grafts, combining osteogenic progenitors with biocompatible scaffolds. For this purpose, bone marrow stromal cells (BMSC) are a rich source of potent osteoprogenitors, able to produce bone tissue both ectopically and orthotopically when associated with ceramic biomaterials (Iaquinta et al., 2019). However, in general this strategy has yet to prove effective in humans for defects of clinically relevant size (i.e., a few cubic centimeters). One significant issue underlying these failures is the lack of rapid vascularization, which results in progenitor death and failure of bone formation inside the graft at depths greater than about 1 mm (Scheufler et al., 2008; Guven et al., 2011).

Several approaches to accelerate vascularization of tissue-engineered bone grafts are currently being investigated, such as surgical techniques (flap or arterio-venous loop fabrication), biomaterial-based methods (scaffold micro-fabrication designed to facilitate vascular in-growth) or co-culture of osteogenic and vasculogenic progenitors inside the grafts (Rao and Stegemann, 2013; Almubarak et al., 2016). We have previously developed biological substitutes of clinically relevant size in a rabbit model by combining bone tissue engineering and flap pre-fabrication (Scheufler et al., 2008). Autologous cell-based, large bone flaps in rabbits were prefabricated using porous ceramic scaffolds loaded with BMSC and wrapped by a panniculus carnosus flap, as an ample exogenous vessel source. This led to the formation of healthy bone tissue in the periphery of the graft, a fibrotic area in the middle and necrosis in the core, because of insufficient blood supply, despite the presence of a highly vascularized panniculus carnosus layer placed around the scaffolds.

Vascular Endothelial Growth Factor (VEGF) is the master regulator of blood vessel growth and the key molecular target of strategies to promote vascular network expansion in regenerative medicine (Gianni-Barrera et al., 2020). Here, we test the hypothesis that increased angiogenic stimulation from within the core of bone constructs by VEGF can significantly accelerate early vascular in-growth, improve graft perfusion and therefore ensure cell viability deeper in the construct.

## Materials and Methods

### Bone Marrow Harvest and rBMSC Culture

The study was approved by the Animal Ethics Committee of the Swiss Federal Veterinary Office^1^. Bone marrow was harvested from 7 young adult New Zealand White (NZW) rabbits (Charles River Laboratories, Kisslegg, Germany) with an average body weight of 2.3 ± 0.2 kg. Animals were sedated by subcutaneous administration of 25 mg per kg of body weight ketamine hydrochloride (Ketaminol 5%^®^ad us. vet., Veterinaria AG, Zurich, Switzerland) and 2.5 mg per kg of body weight xylazine (Narcoxyl 2%^®^ ad us. vet., Veterinaria AG, Zurich, Switzerland) before transfer to the operating room, where inhalation anesthesia was initiated with isoflurane (Forene^®^, Abbott AG, Baar, Switzerland). Transcutaneous arterial oxygen saturation and heart rate were monitored with a pulse oximeter (NPB 290, Nellcor Puritan Bennett, Pleasanton, CA, United States) fixed to a front limb. Before surgery, single shot antibiotic prophylaxis with 15 mg per kg of body weight sulfadoxin-trimethoprim (Borgal^®^ 24% ad us. vet., Veterinaria AG, Zurich, Switzerland) and 0.05 mg per kg of body weight buprenorphine (Temgesic^®^, Essex Chemie AG, Luzern, Switzerland) for pain relief were administered subcutaneously. After the area over the iliac crests was shaved and prepped with povidone iodine solution (Betaseptic^®^, Mundipharma Medical Company, Basel, Switzerland), bone marrow was harvested by repeated puncture of both iliac bones (2–3 per side) using 20 gauge needles and 20 ml syringes filled with 1 ml of heparin sodium solution (Heparin-Na^®^ 5′000 IU/ml, B. Braun Medical AG, Emmenbrücke, Switzerland), yielding average aspirate volumes of 18 ± 4 ml (range: 13–24 ml) per animal. Fresh aspirates were diluted with a double volume of phosphate buffered saline (PBS, Gibco, Invitrogen Corporation, Basel, Switzerland), and centrifuged, resulting in the elimination of supernatant fat, blood clots, and small tissue particles. Nucleated cells were then stained with crystal violet 2.3% (Sigma-Aldrich, Fluka Chemie AG, Buchs, Switzerland), counted and plated at a density of 1 × 10^5^ cells/cm^2^ on tissue culture flasks. *In vitro* culture was performed in α-minimum essential medium (Gibco, Grand Island, NY, United States) supplemented with 10% fetal bovine serum and FGF-2 (5 ng/ml; R&D Systems, Minneapolis, MN, United States). Before reaching cell confluency, rBMSC were detached with 0.05% trypsin/0.01% EDTA (Gibco), counted and replated at a density of 2 × 10^3^ cells/cm^2^ for cell *in vitro* expansion.

### Cell Cycle Analysis

The proportion of actively cycling cells was determined by measuring their nuclear DNA content by flow cytometry after staining with propidium iodide as described before (Helmrich et al., 2012). The data were analyzed using the cell cycle analysis tool from FlowJo Software (Becton, Dickinson and Company) using the Watson model. BMSC from three different rabbits from duplicate dishes were analyzed at each time point.

### Vector Construction

Total RNA was purified from 20 to 30 mg samples of rabbit kidney and spleen tissue with the RNeasy Mini Kit (Qiagen, Basel, Switzerland). cDNA was synthesized with Omniscript Reverse Transcription Kit (Qiagen, Basel, Switzerland) according to the manufacturer’s instructions. Full rabbit VEGF sequence was not available. Watkins et al. described the complete CDS of rabbit VEGF, but they used primers based on human VEGF sequence for the amplification (Watkins et al., 1999). One of the two partial sequences of rbVEGF we found on NCBI matched with the 3′ end of Watkins’ VEGF sequence. The forward primer was constructed based on the human sequence; however, being the leading sequence, it would not interfere with the final amino acid sequence. Full-length rbVEGF-165 was cloned from kidney-RNA by PCR using the following primers: forward: 5′- TTT GGA TCC ATG AAC TTT CTG CTG TCT T -3′; reverse: 5′- TTT CTC GAG TCA CCG CCT CGG CTT GT -3′. PCR conditions were as follows: 94°C × 2 min + (94°C × 30 s + 58°C × 30 s + 72°C × 60 s) × 40 cycles + 72°C × 5 min. A truncated version of rabbit CD4, comprising only the extracellular and transmembrane domains, was generated by inserting a stop codon after codon 424 (NCBI accession number NP_001075782). PCR was performed on spleen cDNA with the following primers: reverse: 5′- CAA TTG TCA TCA CCG GCA CTT GAC ACA G -3′; forward: 5′- GTT TAA ACA TGA ACC GGA GAA TCT ACT -3′. PCR conditions were: 94°C × 2 min + (94° × 40 s + 52° × 40 s + 72° × 60 s) × 5 cycles + (94°C for 40 s, 58°C for 40 s, and 72°C for 60 s) × 30 cycles + 72°C × 5 min.

### BMSC Retroviral Transduction

The amphotropic retrovirus was produced by Phoenix helper-free packaging lines by transient transfection as previously described (Pear et al., 1993). Rabbit BMSCs were infected at high efficiency with the retroviral constructs according to a previously published protocol (Springer and Blau, 1997). In order to achieve the highest efficiency, transduction was carried out few days after plating, when the cells had their highest mitotic activity. rBMSC were cultured in 60-mm dishes and were incubated with retroviral vector supernatants supplemented with 8 μg/mL polybrene (Sigma-Aldrich) for 5 min at 37°C and centrifuged at 1100 *g* for 30 min at room temperature in the dishes, followed by fresh medium replacement. The infection rate of the rBMSC after each transduction round (0 to 6 rounds) was determined by FACS analysis with mouse anti rabbit CD4-FITC conjugated antibody (MCA799F, AbD Serotec).

### Cell Sorting

Expression of truncated CD4 by individual cells was assessed by staining transduced rBMSC with a specific antibody to rabbit CD4 directly conjugated to fluorescein isothiocyanate (MCA799F, AbD Serotec). 3–5 × 10^5^ cells were resuspended into 200 μl of 0.5% BSA and incubated with 5 μl of antibody for 20 min on ice. Data were acquired with a FACS Calibur flow cytometer (Becton, Dickinson and Company) and analyzed using FlowJo software (Becton, Dickinson and Company). Cell sorting was performed with a FACS Vantage SE cell sorter (Becton, Dickinson and Company).

### Rabbit-VEGF ELISA Measurements

The production of rabbit-VEGF in cell culture supernatants was quantified using a Quantikine mouse VEGF immunoassay ELISA kit (R&D Systems Inc., Minneapolis, MN, United States). One ml of fresh medium was incubated on rBMSCs cultured in 60-mm dishes in duplicate for 4 h, filtered, and frozen. Results were normalized by the number of cells in each dish and the time of incubation.

### 3D Perfusion Seeding

Porous ceramic scaffolds (porosity: 80 ± 3%, pore size distribution: 22%, 100 μm; 32%, 100–200 μm; 40%, 200–500 μm; 6%, 500 μm) made of 100% hydroxyapatite, with a Ca/P ratio of 1.66 ± 0.5 (Engipore^®^, Fin-Ceramica, Faenza, Italy^2^), were fabricated in the shape of large tapered cylinders (30 mm height, 20 mm upper base diameter, 10 mm lower base diameter, 5.5 cm^3^ volume). Autologous expanded rBMSC were seeded in tapered cylinders for each animal, using a previously described bioreactor system (Wendt et al., 2003), based on the principle of direct perfusion of a single cell suspension through the interconnected pores of 3D scaffolds. Briefly, scaffolds were pre-wetted in complete medium and press-fitted into custom-made polycarbonate chambers (one scaffold per chamber), positioned at the bottom of two vertical Teflon-columns and connected with each other at their base through a U-shaped tubing. rBMSC (naïve, control CD4 and VEGF-expressing) suspended in 10 ml of medium were introduced into the bioreactor and perfused through the ceramic pores in alternating directions at a flow rate of 1.2 ml/min for 18 h using a standard syringe pump (Programmable PHD 2000^®^, Harvard Apparatus, Holliston, MA, United States). A seeding density of 10 × 10^6^ cells/cm^3^ of ceramic was consistently used, corresponding to a total number of 55 × 10^6^ rBMSC for tapered cylinders. All 3D perfusion cultures were incubated in humidified atmosphere at 37°C/5% CO_2_.

### Surgical Procedure

Cell-seeded ceramic scaffolds were retrieved from the bioreactor, rinsed in PBS, placed in sterile Falcon tubes pre-filled with PBS, and transferred to the operating room. After the surgical site on the dorsum of animals was shaved, disinfected and draped, a midline skin incision was made to expose the panniculus carnosus. Three anteriorly pedicled 6.5-cm wide and 8-cm long panniculus carnosus flap centered over an axial vascular pedicle were raised: two flaps were located cranially and one flap caudally. The cell-scaffold constructs (one with VEGF-transduced BMSC, one with CD4 BMSC and one with naïve BMSC) were randomly assigned to one of the three flap sites, wrapped with a panniculus carnosus flap and covered by a semipermeable membrane (Biobrane^®^, UDL Laboratories Inc., Rockford, IL, United States). The membrane material is permeable to oxygen and nutrients, and allows drainage of exudate through small pores, thereby preventing the accumulation of wound fluid and seroma formation. After 8 weeks, rabbits were euthanized by intravenous injection of pentobarbital (Nembutal^®^, Sanofi, Basel, Switzerland) and constructs retrieved with the surrounding semipermeable membrane. The vascular pedicle was isolated at the flap base and inspected for patency. After division of the vascular pedicle, the constructs were exposed and assessed macroscopically for signs of infection and external vascularization.

### Dynamic Contrast Enhanced MRI

Perfusion MRI was performed on a 3T clinical scanner (Siemens Magnetom Verio) with a 15-channel transmit/receive kneecoil with a dynamic 3D GE sequence (TE 1.38 ms, TR 3.8 s, flip angle 15°, spatial resolution 0.9 × 0.9 × 0.9 mm^3^, dynamic scans sampled every 6 s, total 60 dynamic scans). MRI signal change upon contrast-enhancement was analyzed semi-quantitatively using the initial uprising slope of the signal time curve as a measure of tissue perfusion (”wash-in”) and the initial area under the curve at 20 dynamic scans after contrast arrival (AUC20) as a measure for blood volume fraction after normalization to baseline. Slope and bolus arrival were computed (after manual selection of contrast arrival time on the signal time curves) using in-house MATLAB scripts (MATLAB R2018a, MathWorks^®^).

### Microtomography

After explantation, constructs were fixed in 4% paraformaldehyde overnight and stored in PBS until micro-computed tomography (μ-CT) analysis. Data were acquired by using a phoenix nanotom m scanner (General Electric, Fairfield, CT) with 0.5-mm aluminum filtered x-rays (applied voltage 70 kV; current 260 μA). Transmission images were acquired during a 360° scan rotation with an incremental rotation step size of 0.25°. Reconstruction was made using a modified Feldkamp algorithm at an isotropic voxel size of 2.5 μm. Threshold-based segmentation and 3D measurement analyses (bone mineral density and volume) were performed using ImageJ software (Schneider et al., 2012) with the BoneJ (Doube et al., 2010) and 3D Shape (Sheets et al., 2013) extensions. The threshold employed for the segmentation was set at 325 mg/cm^3^, as previously determined to clearly distinguish between ceramic and newly formed mineralized tissue (Scheufler et al., 2008). Three-dimensional rendering of the structures was performed using VGStudio MAX 2.2 software (Volume Graphics, Heidelberg, Germany).

### Histology and Immunohistochemistry

After μ-CT analysis, constructs were decalcified by 7% EDTA and embedded in paraffin. The sections (7 μm thickness) were stained with Masson’s trichrome staining (RAL Diagnostics, Martillac, France). For immunofluorescence staining, sections were first deparaffinized in Ultra-clear and then rehydrated in graded ethanol. For antigen retrieval, sections were incubated with 0.05% trypsin and 0.1% CaCl2 in water for 13 min at 37°C and 10 min at room temperature, then rinsed 3 × 10 min in 100 mM glycine and 2 × 2 min in PBS. Blocking was performed for 1 h at room temperature with 5% goat serum and 2% BSA in PBS/0.3% Triton. Sections were then incubated for 1 h at room temperature with anti-rabbit CD4 primary antibody (MCA799GA, Clone Ken-4, Bio-Rad, Hercules, CA, United States 1:100) and subsequently for 1 h with a fluorescently labeled secondary antibody (AlexaFluor 647, Invitrogen, Carlsbad, CA, United States 1:200). Fluorescence images were acquired with a Nikon Ti2 Eclipse epifluorescence microscope (Nikon, Tokyo, Japan). The number of CD4^+^ cells was quantified automatically on 5 representative images from each of 3 concentric 1.5 mm-deep layers (outer, middle and inner) in each construct (*n* = 4 constructs/group) using FIJI software (ImageJ^3^) and normalized by the number of total cells (detected by DAPI staining) or by the tissue area (mm^2^). To assess vascularization, immunohistochemical staining for CD31 with a rabbit specific antibody (SC1506, Santa Cruz, 1:100) was performed. After incubation with a biotinylated secondary antibody and subsequently with an ABC-alkaline phosphatase complex, the specific staining was revealed by using Fast Red (all reagents from Dako, Bollschweil, Germany). Matched IgG control antibody was used as a negative control.

### Statistics

Data are presented as mean ± SEM. The normal distribution of all data sets was assessed by the Shapiro-Wilk test and the significance of differences was evaluated with analysis of variance (ANOVA) followed by the Bonferroni test (for multiple comparison), or with the Student’s *t*-test (for single comparisons); *p* < 0.05 was considered statistically significant.

## Results

### Optimization of rBMSC Transduction

**FIGURE 1:**
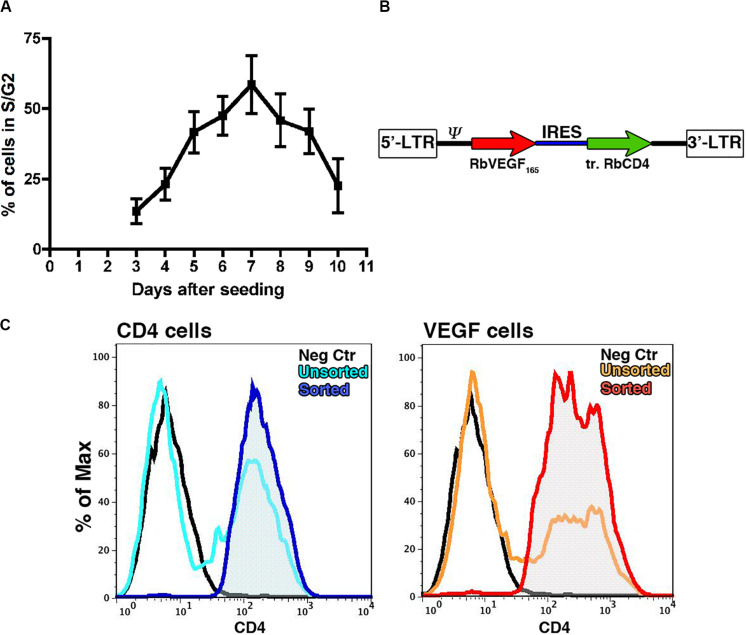
**(A)** Assessment by flow cytometry of the proportion of cells in active proliferation (S/G2 phases) at different time points after initial plating (*n* = 3). **(B)** Map of the bicistronic retroviral vector carrying the coding sequences of rabbit VEGF_165_ (rVEGF_165_) and of a truncated version of rabbit CD4 (tr.rCD4) linked through an Internal Ribosomal Entry Sequence (IRES). **(C)** Representative FACS plots for transduced rBMSC (light blue and orange plots) and FACS purified populations (blue and red plots) stained for CD4 as a marker of transduction; Isotype controls = black plots.

VEGF expression throughout the osteogenic graft was ensured by genetically engineering the seeded progenitors with a retroviral vector (Helmrich et al., 2012), which integrates in the genome and therefore is not lost upon cell expansion. Since retroviral vectors efficiently transduce only dividing cells, we first determined the earliest time after isolation and plating when rabbit BMSC (rBMSC) enter the cell cycle and proliferation is at its peak. Cell cycle analysis was performed on samples from 3 independent donors starting from day 3 after the initial plating to the time of first confluence, which was reached consistently by day 10. rBMSC proliferation increased 5 days after isolation (41.7 ± 7.3% of cycling cells), reproducibly reached a peak by day 7 (58.5 ± 10.3%) and it was maintained until day 9 (41.9 ± 7.9%), after which replication declined with increasing confluence (Figure 1A). Based on these results, we determined the optimal time to start transduction to be on day 6 after isolation. Cells were transduced twice per day up to six times with a retroviral vector expressing a truncated version of rabbit CD4 (trCD4), which was non-functional through removal of the intracellular domain and acted as a convenient cell-surface reporter gene (Misteli et al., 2010). The transduction efficiency was evaluated after each round by FACS quantification of the CD4-positive cells. More than half of the cells were transduced after two rounds (56.7% ± 1.2%) and the number of CD4-positive cells reached 70% after six rounds (70.1 ± 5.2%). Although a plateau was not reached, it was determined that six rounds of transduction would provide a suitable balance between maximizing transduction efficacy and minimizing cell manipulation and this protocol was used to generate all cell populations for further analysis described below.

### Generation of FACS-Sortable VEGF-Expressing rBMSC

A bicistronic retroviral vector (Figure 1B) was constructed carrying the cDNA for rabbit VEGF_165_ (VEGF) and the truncated version of rabbit CD4 (trCD4), joined through an internal ribosomal entry site (IRES) that allows the translation of both proteins from the same mRNA at a fixed ratio (Banfi et al., 2012). A retroviral vector expressing only trCD4 in the second cistron was used to generate control cells. Freshly isolated rBMSC from 4 individual animals were transduced according to the optimized protocol previously described (Helmrich et al., 2012). Using freshly produced viral vector supernatants, the average transduction efficiency was 63.4 ± 20.9% with the VEGF vector and 50.6 ± 16.2% for the control vector (*n* = 4). Upon reaching the first confluence, rBMSC were FACS purified in order to remove non-transduced cells and to yield pure CD4-positive populations (Figure 1C), which were replated for *in vitro* cell expansion. Removal of non-transduced cells was done to ensure a homogeneous distribution of the signal throughout the constructs, because VEGF binds tightly to extracellular matrix and therefore remains highly localized in the microenvironment around each producing cell *in vivo* and does not diffuse through tissue (Ozawa et al., 2004; Gianni-Barrera et al., 2020). Production of VEGF protein by VEGF-expressing rBMSC was confirmed by ELISA (609.5 ± 284.6 ng/10^6^ cells/day of rabbit VEGF), whereas naïve and CD4-rBMSC secreted background amounts (23.5 ± 22.9 and 24.2 ± 25.9 ng/10^6^ cells/day, respectively).

### Generation of the Osteogenic Constructs

**FIGURE 2:**
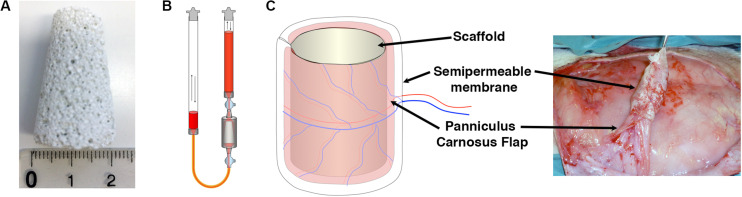
**(A)** Porous ceramic scaffolds were fabricated in the shape of large tapered cylinders (30 mm height, 20 mm lower base diameter, 10 mm upper base diameter, 5.5 cm^3^ volume). **(B)** Schematic representation of the perfusion bioreactor system used in the study, providing bidirectional alternating flow through the construct chamber during cell seeding and culture. **(C)** Constructs were covered with a panniculus carnosus vascularized flap, insulated with a semipermeable membrane and implanted subcutaneously *in vivo*.

Clinical-size osteogenic constructs were generated as previously described (Scheufler et al., 2008) with tapered hydroxyapatite cylinders of 2-cm diameter at the base, 1-cm diameter at the top and 3.5-cm height (Figure 2A). Expanded rBMSC were seeded using a perfusion bioreactor system (Figure 2B), which is based on the principle of direct perfusion of a single cell suspension through the interconnected pores of 3D scaffolds and which has been shown previously to ensure a homogenous cell distribution within the pores of the scaffolds (Wendt et al., 2003). Naïve, VEGF-expressing and CD4-rBMSC were generated from each of the four independent rabbit donors (*n* = 4). Each rabbit received one construct for each condition generated with its own autologous cells. Scaffolds were first wrapped in a panniculus carnosus flap, which provided the vascularization source for new vessels to invade the construct from the outside toward the core, and further covered by a semipermeable membrane and then implanted under the skin of the dorsum of each rabbit donor for 8 weeks (Figure 2C). The membrane employed in this study was used to avoid uncontrolled sources of vascularization besides the panniculus carnosus, since it is permeable to oxygen and nutrients, but does not allow the invasion by surrounding blood vessels and cells. At the moment of harvesting, all constructs appeared viable, surrounded by a healthy panniculus carnosus layer, which was not removed in order to preserve the vascular connections with the scaffolds.

### VEGF-Expressing rBMSC Increase the Amount of Mineralized Tissue Throughout the Construct *in vivo*

**FIGURE 3:**
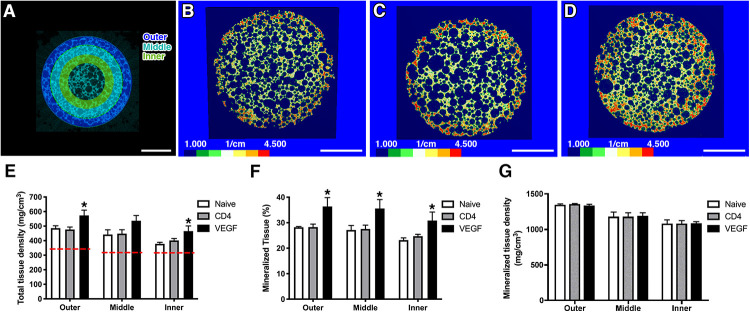
**(A)** For μ-CT analysis, 3 concentric sections, each 1.5-mm deep, were designed in order to distinguish an outer, middle and inner layer (size bar = 5 mm). **(B–D)** Representative images of constructs containing naïve rBMSC **(B)**, CD4- **(C)** and VEGF-expressing rBMSC **(D)**, color-coded according to tissue density (Houndsfield units, HU) (size bar = 5 mm); **(E–G)** Quantification of total tissue density **(E)**, percentage of mineralized tissue **(F)** and density of mineralized tissue **(G)** in the three concentric layers defined in panel **(A)** (*n* = 4, *p < 0.05). The red dashed lines in **(E)** represent the density values of empty scaffolds implanted without any cells.

Constructs were harvested 8 weeks after implantation. Before undergoing decalcification for histological procedures, the constructs were analyzed by micro-computerized tomography (μ-CT). The analysis was performed mid-way on the long axis at a virtual cross-section of 1.5 cm and 3 concentric sections, each 1.5-mm deep, were designed in order to distinguish an outer, middle and inner layer (Figure 3A). Tissue density was calibrated according to Houndsfield units (HU) and color-coded (HU scale, Figures 3B–D). Constructs containing VEGF-expressing rBMSC were characterized by higher HU values reaching deeper in the construct (evidenced by the areas colored in red and orange in Figure 3D) compared to the Naïve (Figure 3B) and CD4 control (Figure 3C) conditions. These data were confirmed by quantification of the total tissue density (Figure 3E). As expected, a gradual decrease in tissue density could be observed at increasing depths from the construct surface. However, VEGF expression induced a consistently greater tissue density in all layers. Interestingly, VEGF enabled the inner layer to reach a tissue density similar or greater than the one of the more superficial layer in control conditions (VEGF inner = 497.4 ± 38.7 mg/cm^3^ vs. Naïve outer = 486.1 ± 28.6 mg/cm^3^ and CD4 outer = 477.0 ± 33.3 mg/cm^3^). The increased tissue density in each layer of the constructs containing VEGF-expressing cells was due to a significant increase by about 25% in the amount of mineralized tissue compared to the control conditions (Figure 3F). On the other hand, the density of the mineralized tissue itself was not different among the three conditions (Figure 3G), showing that VEGF did not affect the quality of the mineralized matrix, but enabled a greater amount of it to be deposited.

### VEGF Expression Increases Dense Tissue Ingrowth

**FIGURE 4:**
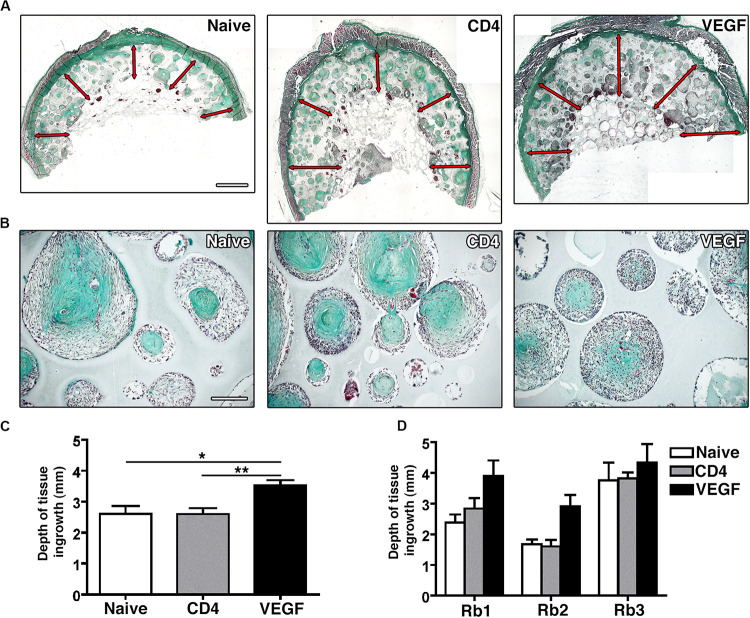
**(A)** Representative macroscopic pictures of graft sections stained with Masson’s Trichome. Red arrows indicate tissue invasion (size bar = 2 mm). **(B)** Representative pictures of scaffold pores containing collagenous dense matrix (Masson’s trichrome staining, size bar = 100 μm). **(C)** Quantification of the average depth of tissue ingrowth for the 3 conditions (*n* = 3, *p < 0.05, **p < 0.01). **(D)** Quantification of the depth of tissue ingrowth for the three individual rabbits (Rb1-3) included in the overall analysis in panel **(C)**.

Tissue ingrowth and bone formation were assessed histologically by Masson’s trichrome staining. Constructs loaded with naïve, control CD4 and VEGF-expressing rBMSC were analyzed at three different positions along the main axis of the constructs in three independent rabbits and tissue invasion was quantified on transverse sections at five standardized points within each transverse section (red arrows in Figure 4A). Frank bone tissue could not be detected in any condition, but dense collagenous matrix could be observed to fill the scaffold pores in all conditions, similar to pre-bone matrix not yet fully mineralized (Figure 4B). However, the depth of growth of this dense tissue was significantly different among conditions (Figure 4C). In fact, while both naïve and control CD4 cells similarly generated dense tissue in about the first 2.5 mm from the construct surface, VEGF-expressing rBMSC promoted a 35% increase in the depth of tissue invasion (3.52 ± 0.17 mm vs. naïve = 2.6 ± 0.25 mm and CD4 = 2.59 ± 0.20 mm; *p* < 0.05 and <0.01, respectively). A separate analysis of the data confirmed the reproducibility of this trend in the individual rabbits (Figure 4D).

### VEGF Expression Improves the Survival of Seeded rBMSC

**FIGURE 5:**
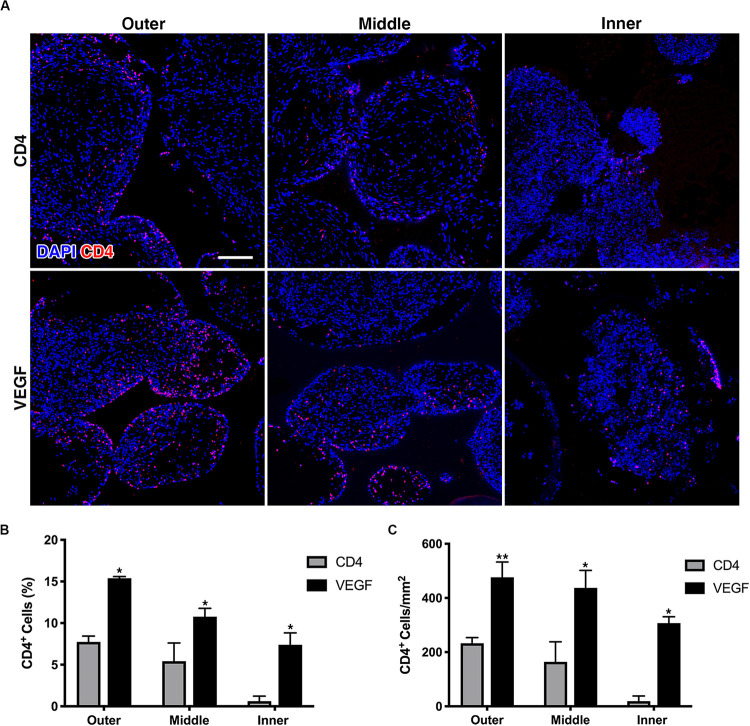
**(A)** Representative immunofluorescence images from the outer, middle and inner layers of graft sections stained for rbCD4 (red) to detect the seeded progenitors and counterstained with DAPI (blue) to label nuclei (size bar = 100 μm); **(B,C)** quantification of the number of CD4^+^ cells, expressed as percentage (%) of the total cell number **(B)** and as absolute number per mm^2^ of tissue area **(C)** in each layer (*n* = 4, *p < 0.05, **p < 0.01).

The survival of seeded progenitors was tracked by immunofluorescent staining of the truncated CD4 marker expressed from the retroviral vectors used to transduce both the control CD4 and VEGF populations, which had been used also for the FACS purification. The quantification of progenitor survival was performed separately in 3 concentric sections, each 1.5-mm deep, in order to distinguish an outer, middle and inner layer, similarly to the analysis of the μ-CT data above. As shown in Figure 5A, rBMSC could be detected within the scaffold pores in both groups 8 weeks after implantation. Quantification of CD4 + cells showed that VEGF expression significantly improved the survival of seeded progenitors by greater than 2-fold in the outer and middle layer, both in relation to the total amount of cells within the pores (Figure 5B) and as absolute amount of cells per mm^2^ of pore tissue (Figure 5C). However, the most striking difference was observed in the inner layer, where progenitor survival dropped to virtually zero in the controls, but was maintained at levels similar to the middle layer by VEGF expression (Figure 5C; Outer: CD4 = 232.6 ± 21.2 cells/mm^2^ vs. VEGF = 476.1 ± 56.7 cells/mm^2^, *p* < 0.01; Middle: CD4 = 164.4 ± 73.7 cells/mm^2^ vs. VEGF = 437.1 ± 64.7 cells/mm^2^, *p* < 0.05; Inner: CD4 = 19.1 ± 19.1 cells/mm^2^ vs. VEGF = 307.1 ± 23.9 cells/mm^2^, *p* < 0.05).

### VEGF-Expressing rBMSC Improve Vascularization to the Core and Promote Artery Formation in the Periphery

**FIGURE 6:**
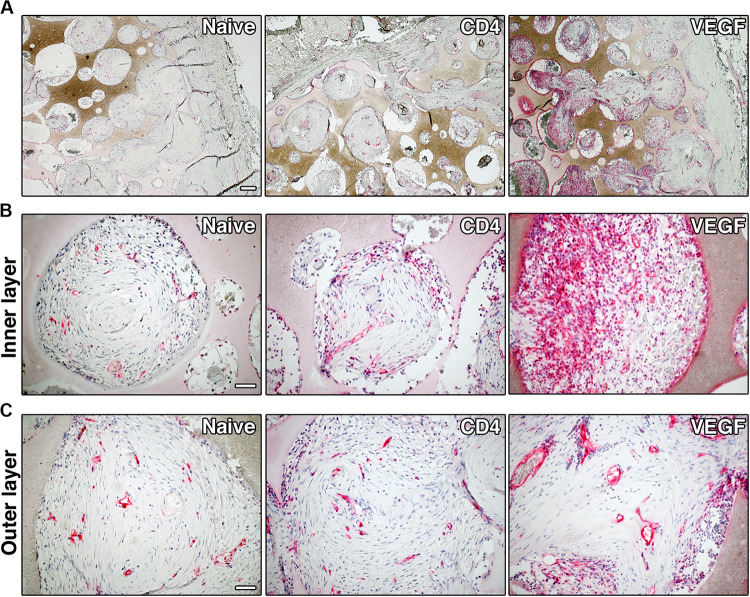
Representative images of graft sections immuno-stained for CD31, showing blood vessels (in red), at low-magnification (**A**, size bar = 200 μm) and high-magnification (**B**, inner layer and **C**, outer layer, size bar = 50 μm).

In order to determine whether better progenitor survival and tissue growth could be consequent to improved vascularization of the VEGF-expressing constructs, blood vessels were visualized histologically by CD31 staining (Figure 6). By the end of the experiment 8 weeks after implantation, some blood vessels were present in most of the pores of the scaffolds in all three conditions (red signal in Figure 6A). However, the VEGF-expressing scaffolds were invaded by much more abundant and denser vascular networks throughout the depth of the constructs. Moreover, VEGF overexpression promoted the formation of dense micro-vascular capillary networks in the deeper areas (Figure 6B), whereas both control conditions contained only rare small-caliber vessels within the deep pores. Interestingly, the outer shell of VEGF constructs was vascularized by large-caliber vessels, with homogenous diameters and filled with red blood cells, corresponding to arteries and veins, whereas the morphology and frequency of vessels in the naïve and CD4 constructs was similar between the outer and inner regions (Figure 6C).

### VEGF-Expressing rBMSC Improve the Depth of Functional Early Blood Perfusion

**FIGURE 7:**
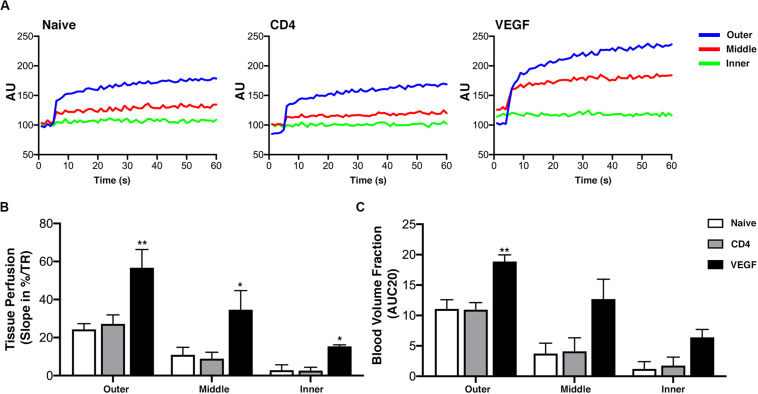
Early blood flow inside the constructs assessed non-invasively by dynamic contrast-enhanced magnetic resonance imaging (DCE MRI) at 1 week after implantation (*n* = 4). **(A)** DCE-MRI signal time-course for each layer in naïve, CD4 and VEGF-expressing constructs, expressed in arbitrary units (AU). **(B)** Tissue perfusion computed from contrast “wash-in,” i.e., the initial uprising slope of the signal-time curve, expressed as the percentage increase over repetition time (%/TR). **(C)** Blood volume fraction determined from the initial area under the normalized DCE curve at 20 dynamic scans after contrast arrival (AUC20) for each layer and construct (*p < 0.05, **p < 0.01).

The histological data on tissue growth and blood vessel formation suggest that the beneficial effect of VEGF expression may take place by improving blood flow to critically under-perfused progenitors in the middle layer of the constructs, thereby enabling their survival and differentiation. To verify this hypothesis, early blood flow inside the constructs was measured non-invasively by dynamic contrast enhancement in the MRI at the crucial time of 1 week after implantation. The analysis was performed similarly to the μ-CT data. Tomographic images were selected mid-way on the long axis at a virtual cross-section of 1.5 cm and three concentric sections, each 1.5-mm deep, were designed in order to distinguish an outer, middle and inner layer. MRI signal intensity upon contrast agent injection was analyzed in each layer over time (60 dynamic scans, Figure 7A) and used to calculate tissue perfusion (Figure 7B) and the blood volume fraction (Figure 7C; Cuenod and Balvay, 2013). The results show that both tissue perfusion and blood volume fraction were significantly increased in the outer and middle layers of VEGF-expressing constructs compared to naïve and CD4 controls. Interestingly, VEGF expression improved blood flow in the middle layer to similar levels as in the outer layer of the controls. Tissue perfusion was improved also in the inner layer by VEGF, but it only reached levels similar to the middle layer of the controls.

## Discussion

In this study, we show that sustained VEGF over-expression in clinical-size osteogenic grafts by genetically modified rabbit BMSC was effective to significantly: (1) increase the depth of tissue ingrowth; (2) improve the long-term survival of seeded progenitors and the formation of mineralized tissue; (3) support the establishment of a hierarchical vascular network, and (4) improve early blood perfusion in clinical-size scaffolds.

Even though the initial growth of new vessels can be quite rapid, sustained VEGF signaling for about 4 weeks is required for their subsequent stabilization and ability to persist indefinitely. In fact, if delivery is too transient, newly induced vessels regress promptly upon cessation of the VEGF stimulus (Gianni-Barrera et al., 2020). Therefore, rapid passive release of recombinant factors from scaffolds is inadequate to ensure new vessel persistence and a large body of work has focused on different strategies to ensure controlled and sustained delivery of angiogenic molecules (Martino et al., 2015). VEGF over-expression in BMSC was previously shown to ensure sustained angiogenic signaling and to promote vascularization in non-clinical size osteogenic grafts implanted subcutaneously in nude rats (Helmrich et al., 2013). However, it is not clear whether such growth could be sufficiently fast and extensive to reach the tissue depths required in clinical-size constructs, where the rapid establishment of a vascular network is needed to ensure progenitor cell survival in the core of the scaffold. In fact, survival of the implanted cells in small constructs is supported by the availability of surrounding vasculature within a short distance. On the contrary, in constructs of clinically relevant size (i.e., a few cubic centimeters) the distance for diffusion of oxygen and nutrients is exceeded and vessel in-growth depends on physiological VEGF upregulation by ischemic conditions generated inside the construct. However, this process is too slow to ensure timely vascularization to significant depths, causing cell death after transplantation and failure of tissue formation (Sharma et al., 2019). We previously investigated the potential of providing a highly vascularized tissue source around the constructs (Scheufler et al., 2008). However, the simple availability of abundant vascular networks around the graft still requires endogenous upregulation of angiogenic signals induced by ischemia to recruit new vessel growth inside the construct. Therefore, bone formation was still limited to the outer periphery and could not exceed about 1 mm of depth, while only fibrous tissue was formed more deeply. Here, we tested the strategy to combine a rich vascular supply around the implants (*panniculus carnosus*) with angiogenic signals directly produced by the osteogenic progenitors seeded throughout the scaffolds in constructs of clinically relevant size (5.5 cm^3^). To this end, rabbit bone marrow mesenchymal progenitors were genetically modified with a retroviral vector to stably overexpress VEGF and ensure consistent long-term expression despite cell expansion (Helmrich et al., 2012). We have previously reported that *in vivo* delivery of retrovirally transduced progenitors producing therapeutic and functional levels of VEGF led to an increase in tissue VEGF protein at least 6-fold higher than that induced by ischemia alone (von Degenfeld et al., 2006). Indeed, our data show that providing a VEGF signal throughout the construct, independently of intervening hypoxia, promoted both the speed and efficacy of vascularization of clinical-size constructs.

Crucially, the functional perfusion of inner layers was significantly improved already by 1 week after implantation. In fact, given sufficient time, all constructs are eventually invaded by vessels to the inner layer (e.g., see Figure 5 after 8 weeks), but perfusion within the first week determines whether seeded progenitors survive (Rouwkema and Khademhosseini, 2016) and recent data suggest that significant progenitor death may start as soon as 3 days after implantation (Moya et al., 2018). The fact that VEGF expression on one hand improved long-term progenitor survival up to 8 weeks, and on the other increased functional perfusion all the way to the inner layer several millimeters inside the constructs within the first 7 days (Figure 7B) suggests that vascular ingrowth can be a rather rapid process, given the appropriate stimulation.

The functional significance of this increase in perfusion can be understood by comparing its distribution within the constructs with that of tissue formation. Under control conditions (Naïve and CD4 cells) tissue formation took place to a depth of about 2.5 mm (Figure 4C), indicating that blood perfusion is required at a level between that of the outer and middle layers (depths of 0–1.5 and 1.5–3.0 mm, respectively), whereas the very little flow in the inner layer (3.0–4.5 mm depth) is incompatible with progenitor survival, which in fact were essentially absent in the inner layer of control grafts (Figure 5). VEGF expression increased perfusion in all three layers, leading the middle and inner layers to receive a similar blood flow as the outer and middle layers of the control conditions, respectively (Figure 7). Consistently with these changes, the survival of seeded progenitors was significantly improved by VEGF expression all the way through the inner layer and tissue formation was promoted throughout the middle layer and into the inner layer, to about 3.5 mm of depth. Although blood flow in the outer layer of controls was already sufficient to enable tissue formation, the increase due to VEGF expression appeared to be beneficial also in this location, as evidenced by the improvement in tissue density and mineralization (Figures 3E,F).

The accelerated kinetics of vessel growth afforded by VEGF over-expression is also reflected in the different structure of the definitive vascular networks visible after 8 weeks (Figure 6). The slow growth in the naïve and CD4 conditions yielded only a sparse microvascular network, with similar morphology throughout the constructs. On the other hand, the robust and rapid growth induced by sustained VEGF signaling led to a hierarchically organized functional vascular tree, composed of dense capillary networks in the inner layers, capable of ensuring a robust exchange of nutrients and respiratory gases, connected to large-caliber feeding vessels entering the constructs at the periphery, which are necessary to supply effective blood flow to the inner layers. In fact, the effective expansion of the microvascular capillary bed by VEGF (angiogenesis) has been shown to induce enlargement and recruitment of feeding arteries (arteriogenesis) (Springer et al., 2003) both through increased shear stress and gap junction-mediated retrograde signaling along the vessel walls (Pries et al., 2010; Annex, 2013).

One limitation emerging from this study is that osteogenic differentiation did not reach the stage of frank bone. In all groups the tissue that was formed displayed histological features typical of osteogenic commitment of progenitors and clearly distinct from fibrous tissue, namely the deposition of a dense and homogeneous collagenous matrix, within which sparse cells remained embedded (Figure 4B). Further, μ-CT analysis showed density values typical of a mineralized matrix (500–600 mg/cm^3^ in the VEGF conditions; Figure 3E) and denser than both the HA scaffold measured as a reference (<325 mg/cm^3^) and the same scaffolds implanted without any cells (red dashed lines in Figure 3E), thereby ruling out the possibility of pathological calcification. This incomplete osteogenic differentiation was not due to VEGF expression or the genetic modification of the progenitors, as both VEGF and CD4 cells behaved similarly to the naïve condition. Rather, the need to introduce a FACS-purification step after retroviral transduction reduced the amount of available rBMSC and required their expansion for >18 population doublings in order to obtain sufficient autologous cells for each rabbit to seed 3 clinical-size constructs. Although naïve cells did not require FACS purification, they underwent the same degree of *in vitro* expansion in order to maintain appropriate control conditions. In fact, it is well known that BMSC dramatically lose their *in vivo* bone-forming capacity during the first few passages of *in vitro* expansion (Banfi et al., 2000). The importance of limiting BMSC *in vitro* culture can be appreciated by comparing the results of previous experiments in which the same constructs were seeded with the same amount of naïve rBMSC expanded only until the first confluence (about 12 population doublings), where frank bone formation was observed in 9/12 constructs (75%), albeit only to a depth of <1 mm (Scheufler et al., 2008).

In conclusion, these data constitute proof of principle that providing sustained VEGF signaling, independently of cells experiencing hypoxia, is effective to drive rapid vascularization and increase early perfusion in clinical-size osteogenic grafts, leading to improved progenitor survival and tissue formation deeper in the constructs. However, when VEGF delivery is achieved by genetic modification of the osteoprogenitors, the necessary degree of *in vitro* expansion can reduce their *in vivo* bone-forming capacity. Therefore, it would be desirable to achieve sustained VEGF delivery in the constructs while limiting *in vitro* manipulation of the osteoprogenitors. One strategy could be to optimize further the genetic modification process to achieve greater transduction efficiencies and avoid the FACS purification step to eliminate non-transduced cells. In fact, retroviral vectors infect only dividing cells, with greater efficacy the faster the cell cycle (Springer and Blau, 1997), and rBMSC transduction was limited to 70% despite targeting the protocol to the proliferation kinetics. However, lentiviral vectors infect target cells regardless of cell cycle status and allow more efficient transduction rates (High and Roncarolo, 2019). Further, clinically compliant new generation vectors, such as self-inactivating lentiviruses with chromatin insulator elements providing a more attractive safety profile, as they ensure stable and sustained transgene expression without risks of insertional mutagenesis (De Ravin et al., 2016).

Another attractive strategy would be to avoid genetic modification of progenitors altogether and to provide angiogenic signaling by decorating the scaffold itself with recombinant VEGF protein. For example, a variety of protein engineering approaches have been developed to allow growth factor cross-linking into fibrin matrices or to endow them with super-affinity for extracellular matrix, effectively ensuring that morphogenic signals are both protected from proteolytic degradation and presented to their target cells in the physiological context of matrix association (Martino et al., 2015).

## Data Availability Statement

The datasets generated for this study are available on request to the corresponding author.

## Ethics Statement

The animal study was reviewed and approved by the Animal Ethics Committee of the Swiss Federal Veterinary Office.

## Author Contributions

RL, AS, DS, AB, and ND designed the study. RL, MB, OH, CW, AG, CS, AK, SG, GJ, and ND performed the experiments, acquired, analyzed, and interpreted the data. RL, MB, OH, CW, AG, CS, AK, SG, GJ, AS, DS, AB, and ND wrote/revised the manuscript and gave final approval. All authors contributed to the article and approved the submitted version.

## Conflict of Interest

CS is currently employed by the company Novartis. The remaining authors declare that the research was conducted before such employment and in the absence of any commercial or financial relationships that could be construed as a potential conflict of interest.

## Footnotes

1 http://bvet.admin.ch

2 www.finceramica.it

3 http://fiji.sc/Fiji
